# Supplementary material for: Improving Hepatocellular Carcinoma Surveillance Outcomes in Patients with Cirrhosis after Hepatitis C Cure: A Modelling Study
Source: Cancers (Basel). 2024 Aug 1;16(15):2745. doi: 10.3390/cancers16152745 (PMC11312194; doi:10.3390/cancers16152745)
Supplement: Supplementary file 1 [file cancers-16-02745-s001.zip › cancers-3104257-supplementary.pdf]

Improving Hepatocellular Carcinoma Surveillance Outcomes in Patients with Cirrhosis after Hepatitis C Cure: A Modelling Study

Jacob Cumming [1,2,*] , Nick Scott [1,3], Jessica Howell [1,4,5], Joan Ericka Flores [4] , Damian Pavlyshyn [1],
Margaret E. Hellard [1,3,6,7], Leon Shin-han Winata [4], Marno Ryan [4], Tom Sutherland [4,5], Alexander J. Thompson [4,5],
Joseph S. Doyle [1,6,†] and Rachel Sacks-Davis [1,3,†]

1 Disease Elimination Program, Burnet Institute, Melbourne, VIC 3004, Australia

2 Population Health and Immunity, Walter and Eliza Hall Institute, Parkville, VIC 3052, Australia

3 Department of Epidemiology and Preventive Medicine, Monash University, Melbourne, VIC 3004, Australia

4 Department of Gastroenterology, St Vincent's Hospital, Melbourne, VIC 3065, Australia

5 Department of Medicine, University of Melbourne, Melbourne, VIC 3052, Australia

6 Department of Infectious Diseases, The Alfred and Monash University, Melbourne, VIC 3004, Australia

7 Doherty Institute and School of Population and Global Health, University of Melbourne,

Melbourne, VIC 3052, Australia

* Correspondence: jcacademicresearch@gmail.com

† These authors contributed equally to this work.

June 28, 2024

1 Notation Used in the Supplementary Material

For the notation used in the additional material see Supplementary Table S1. The likelihood is defined in the usual way, that is $\mathcal{L}_{y_1,\dots,y_k}(\theta_1,\dots,\theta_n) := \Pr(y_1,\dots,y_k|\theta_1,\dots,\theta_n)$, and $\text{Binom}(y; n, \theta) := \binom{n}{y}\theta^y(1-\theta)^{n-y}$

Supplementary Table S1: Variables referred to in the Additional Material.

Notation	Range	Description
X	$\{0, \text{A}, \text{B}, \text{C}, \text{D}\}$	BCLC stage of liver cancer.
$p_X, \text{Sym}_X, \text{Sens}_X$	$[0, 1]$	Underlying (population level) probabilities to be estimated.
n_X	$\mathbb{N}$	Number of untreated patients with initial stage X diagnosis as reported in Giannini et al. [1]
m_X	$\mathbb{N}$	Median survival time for untreated patients with initial stage X diagnosis as reported in Giannini et al. [1]
k		Number of regular surveillance patients who developed cancer as reported in Khalili et al. [2]
D_0, D_A	$\{0, \dots, k\}$	Number of regular surveillance patients who were diagnosed in BCLC stage 0 and A respectively, as reported in Khalili et al. [2]
$\bar{s}$	$\mathbb{N}$	Number of liver cancer diagnoses for those not participating in surveillance as reported in Hong et al. [3]
D_α	$\{1, \dots, \bar{s}\}$	Number of liver cancer diagnoses of BCLC stage A/B among for those not participating in surveillance as reported in Hong et al. [3]
$\widehat{p}_X, \widehat{\text{Sym}}_X, \widehat{\text{Sens}}_X$	$[0, 1]$	Maximum likelihood estimates of p_X, Sym_X, and Sens_X with respect to $m_0, m_\text{A}, m_\text{B}, m_\text{C}, m_\text{D}, D_0, D_\text{A}, D_\alpha$.
$m_X^{*b}, D_0^{*b}, D_\text{A}^{*b}, D_\alpha^{*b}$	Same as non-bootstrap	bth Parametric bootstrap samples of $m_X, D_0, D_\text{A}, D_\alpha$.
$\widehat{p}_X^{*b}, \widehat{\text{Sym}}_X^{*b}, \widehat{\text{Sens}}_X^{*b}$	$[0, 1]$	ith maximum likelihood estimates of p_X, Sym_X, and Sens_X using the parametric bootstrap samples m_X^{*b}'s, $D_0^{*b}, D_\text{A}^{*b}$, and D_α^{*b}.

2 The Bootstrap

2.1 Bootstrapping Methods

The model parameters $p_0, p_\text{A}, p_\text{B}, p_\text{C}, p_\text{D}, \text{Sens}_0, \text{Sens}_\text{A}, \text{Sym}_\text{A}$, and Sym_B found using the maximum likelihood inference. The parametric bootstrap was used on the data that the inference was done on, in order to assess the uncertainty of the maximum likelihood estimates.

2.1.1 Data

From Giannini et al. [1],

$$(n_0, n_\text{A}, n_\text{B}, n_\text{C}, n_\text{D}) = (12, 101, 139, 138, 210)$$

and

$$(m_0, m_{\mathrm{A}}, m_{\mathrm{B}}, m_{\mathrm{C}}, m_{\mathrm{D}}) = (38, 25, 10, 7, 4).$$

From Khalili et al. [2],

$$(k, D_0, D_A) = (109, 36, 51).$$

From Hong et al. [3], $\bar{s} = 158$. 75 out of the 127 participants in the study diagnosed with BCLC stage A/B cancer were in regular surveillance. Therefore $D_\alpha = 127 - 75 = 52$.

All three of these studies used different cohorts, and so the reported data sets are assumed to be independent.

2.1.2 Hypogeometric Distribution

The hypogeometric distribution[1] is the distribution of a sum of independent geometric random variables, making it the discrete analogue of the hypoexponential distributions. In other words, $Y \sim \mathrm{HypoGeom}(p_1, p_2, \ldots, p_k)$ if

$$Y \overset{\mathrm{d}}{=} Y_1 + Y_2 + \cdots + Y_k,$$

where $Y_i \sim \mathrm{Geom}(p_i)$ are independent random variables with $p_i \in (0, 1]$.

2.1.3 Likelihood Functions

The number of months until death from stage X is distributed according to

$$\mathrm{HypoGeom}(p_{\mathrm{D}}, p_{\mathrm{C}}, \ldots, p_X).$$

Particularly, time until death from stage D is distributed $\mathrm{HypoGeom}(p_{\mathrm{D}}) = \mathrm{Geom}(p_{\mathrm{D}})$. The likelihoods

$$\mathcal{L}_{m_0}(p_0, p_{\mathrm{A}}, \ldots, p_{\mathrm{D}}), \mathcal{L}_{m_{\mathrm{A}}}(p_{\mathrm{A}}, \ldots, p_{\mathrm{D}}), \ldots, \mathcal{L}_{m_{\mathrm{D}}}(p_{\mathrm{D}})$$

were derived using the distribution of the median order statistic for hypogeometric distribution, with the dOrder function from the DescTools [5] package in R.[2]

Let d_0 be the event that an individual who is completely adherent to liver cancer surveillance recommendations and develops liver cancer is diagnosed in BCLC stage 0.

[1] The hypogeometric distribution is proposed and described more fully in Chukova et al. [4]

[2] Due to limitations with the dOrder function, for medians derived from an even number of observations, we subtracted one from the number of reported observations. For example, m_0 was derived from 12 observations, but we used the likelihood that m_0 was the sixth order statistic from 11 observations.

$$
\begin{aligned}
\Pr(d_0) &= \sum_{j=0}^{5} \Pr(d_0 \text{ and developed cancer } j \text{ months from next US}) \\
&= \sum_{j=0}^{5} \Pr(\text{Developed cancer } j \text{ months from next US}) \Pr(d_0 | \text{Next ultrasound in } j \text{ months}) \\
&= \frac{1}{6} \sum_{j=0}^{5} \Pr(d_0 | \text{Next ultrasound in } j \text{ months}) \\
&= \frac{1}{6} \sum_{j=0}^{5} \Pr(\text{Stay in stage 0 for a month})^j \times \Pr(d_0 | \text{Next ultrasound in zero months}) \\
&= \frac{1}{6} \sum_{j=0}^{5} (1-p_0)^j \\
&\quad \times \sum_{i=0}^{\infty} \Pr(\text{US diagnoses BCLC 0}) \Pr(\text{US misses BCLC 0})^i \Pr(\text{Stay in stage 0 for a month})^{6i} \\
&= \frac{\text{Sens}_0}{6} \sum_{j=0}^{5} (1-p_0)^j \times \sum_{i=0}^{\infty} ((1-\text{Sens}_0) \times (1-p_0)^6)^i \\
&= \frac{\text{Sens}_0}{6} \times \frac{1-(1-p_0)^6}{p_0} \times \frac{1}{1-(1-\text{Sens}_0) \times (1-p_0)^6} \\
&= \frac{\text{Sens}_0 (1-(1-p_0)^6)}{6 \times p_0 (1-(1-\text{Sens}_0)(1-p_0)^6)}. \qquad \text{(Exact Solution)}
\end{aligned}
$$

Therefore

$$
\mathcal{L}_{D_0}(p_0, \text{Sens}_0) = \text{Binom}\left(D_0; k, \frac{\text{Sens}_0 (1-(1-p_0)^6)}{6 \times p_0 (1-(1-\text{Sens}_0)(1-p_0)^6)}\right).
$$

In order to calculate the likelihood relating to D_A, we constructed a transition matrix

$$
T(p_0, p_\text{A}, \text{Sens}_0, \text{Sens}_\text{A})
$$

(see the transition matrix (1) at the end of these materials), with corresponding states as described in Supplementary Table S2

The initial distribution of the Markov chain is

$$
\mathbf{t}^{(0)} = \begin{bmatrix} \frac{\text{Sens}_0}{6} & 0 & \frac{1-\text{Sens}_0}{6} & \frac{1}{6} & \frac{1}{6} & \frac{1}{6} & \frac{1}{6} & \frac{1}{6} & 0 & 0 & 0 & 0 & 0 & 0 & 0 \end{bmatrix},
$$

since we assumed that people have an even probability of developing cancer one to six months out from their next ultrasound. Note that $\mathbf{t}_1^{(0)} = \frac{\text{Sens}_0}{6}$ since there is a probability of $\frac{\text{Sens}_0}{6}$ that people are diagnosed in the same month they develop cancer. The limiting distribution

$$
\mathbf{t}(p_0, p_\text{A}, \text{Sens}_0, \text{Sens}_\text{A}) := \lim_{n \to \infty} \mathbf{t}^{(0)} \times T(p_0, p_\text{A}, \text{Sens}_0, \text{Sens}_\text{A})^n
$$

was numerically approximated by

$$
\mathbf{t}(p_0, p_\text{A}, \text{Sens}_0, \text{Sens}_\text{A}) \approx \mathbf{t}^{(100)}(p_0, p_\text{A}, \text{Sens}_0, \text{Sens}_\text{A}) := \mathbf{t}^{(0)} T(p_0, p_\text{A}, \text{Sens}_0, \text{Sens}_\text{A})^{100}.
$$

Supplementary Table S2: States of the transition matrix 1.

State	Diagnosed	Stage	Months Until Ultrasound
1	Yes	BCLC 0	NA
2	Yes	BCLC A	NA
3	No	BCLC 0	Six
4	No	BCLC 0	Five
5	No	BCLC 0	Four
6	No	BCLC 0	Three
7	No	BCLC 0	Two
8	No	BCLC 0	One
9	No	BCLC A	Six
10	No	BCLC A	Five
11	No	BCLC A	Four
12	No	BCLC A	Three
13	No	BCLC A	Two
14	No	BCLC A	One

Therefore $\mathbf{t}^{(100)}(p_0, p_\mathrm{A}, \mathrm{Sens}_0, \mathrm{Sens}_\mathrm{A})_1$ is approximately the probability of being diagnosed in BCLC stage 0 HCC.[3] Therefore

$$\mathcal{L}_{D_\mathrm{A}|D_0}(p_0, p_\mathrm{A}, \mathrm{Sens}_0, \mathrm{Sens}_\mathrm{A}) = \mathrm{Binom}\left(D_\mathrm{A}; k - D_0, \frac{\mathbf{t}(p_0, p_\mathrm{A}, \mathrm{Sens}_0, \mathrm{Sens}_\mathrm{A})_2}{1 - \mathbf{t}(p_0, p_\mathrm{A}, \mathrm{Sens}_0, \mathrm{Sens}_\mathrm{A})_1}\right),$$

and hence

$$\mathcal{L}_{D_0,D_\mathrm{A}}(p_0, p_\mathrm{A}, \mathrm{Sens}_0, \mathrm{Sens}_\mathrm{A}) = \mathcal{L}_{D_0}(p_0, \mathrm{Sens}_0) \times \mathcal{L}_{D_\mathrm{A}|D_0}(p_0, p_\mathrm{A}, \mathrm{Sens}_0, \mathrm{Sens}_\mathrm{A})$$

Let d_α be the event that an individual who does not participate in surveillance and develops liver cancer is diagnosed with BCLC stage A or B cancer (presumably through symptoms). There is an assumption that symptoms do not develop in stage 0.

[3] To check convergence, we checked the discrepancy of $\mathbf{t}^{(100)}(p_0, p_\mathrm{A}, \mathrm{Sens}_0, \mathrm{Sens}_\mathrm{A})_1$ with the equation (Exact Solution).

$$
\begin{aligned}
\Pr(d_\alpha) &= \Pr(\text{Getting symptoms in stage A}) \\
&\quad + \Pr(\text{Getting symptoms in stage B, and not stage A}) \\
\Pr(\text{Getting symptoms in stage A}) &= \text{Sym}_\text{A} \sum_{i=0}^{\infty} (1-p_\text{A})^i {\text{Sym}_\text{A}}^i \\
&= \frac{\text{Sym}_\text{A}}{1-(1-p_\text{A})\text{Sym}_\text{A}} \\
\Pr\begin{pmatrix}\text{Getting symptoms in stage B,} \\ \text{and not stage A}\end{pmatrix} &= \underbrace{\left(1 - \frac{\text{Sym}_\text{A}}{1-(1-p_\text{A})\text{Sym}_\text{A}}\right)}_{\Pr(\text{No symptoms in stage A})} \times \text{Sym}_\text{B} \sum_{i=0}^{\infty} (1-p_\text{B})^i {\text{Sym}_\text{B}}^i \\
&= \left(1 - \frac{\text{Sym}_\text{A}}{1-(1-p_\text{A})\text{Sym}_\text{A}}\right) \times \frac{\text{Sym}_\text{B}}{1-(1-p_\text{B})\text{Sym}_\text{B}} \\
\implies \Pr(d_\alpha) &= \left(1 - \frac{\text{Sym}_\text{A}}{1-(1-p_\text{A})\text{Sym}_\text{A}}\right) \times \frac{\text{Sym}_\text{B}}{1-(1-p_\text{B})\text{Sym}_\text{B}} \\
&\quad + \frac{\text{Sym}_\text{A}}{1-(1-p_\text{A})\text{Sym}_\text{A}}
\end{aligned}
$$

Therefore

$$
\begin{aligned}
&\mathcal{L}_{D_\alpha}(p_\text{A}, p_\text{B}, \text{Sym}_\text{A}, \text{Sym}_\text{B}) = \\
&\text{Binom}\left(D_\alpha; \bar{s}, \left(1 - \frac{\text{Sym}_\text{A}}{1-(1-p_\text{A})\text{Sym}_\text{A}}\right) \times \frac{\text{Sym}_\text{B}}{1-(1-p_\text{B})\text{Sym}_\text{B}} + \frac{\text{Sym}_\text{A}}{1-(1-p_\text{A})\text{Sym}_\text{A}}\right).
\end{aligned}
$$

This likelihood is the only likelihood that contains Sym_A and Sym_B, so there is no unique maximum likelihood estimator. In other words, the model is non-identifiable. Therefore we set $8 \times \text{Sym}_\text{A} = \text{Sym}_\text{B}$,[4] and we use the likelihood

$$
\begin{aligned}
&\mathcal{L}_{D_\alpha}(p_\text{A}, p_\text{B}, \text{Sym}_\text{A}) = \\
&\text{Binom}\left(D_\alpha; \bar{s}, \left(1 - \frac{\text{Sym}_\text{A}}{1-(1-p_\text{A})\text{Sym}_\text{A}}\right) \times \frac{8 \times \text{Sym}_\text{A}}{1-8(1-p_\text{B}) \times \text{Sym}_\text{A}} + \frac{\text{Sym}_\text{A}}{1-(1-p_\text{A})\text{Sym}_\text{A}}\right).
\end{aligned}
$$

So our final objective function to maximise is

$$
\mathcal{L} := \mathcal{L}_{m_0, m_\text{A}, m_\text{B}, m_\text{C}, m_\text{D}, D_0, D_\text{A}, D_\alpha}(p_0, p_\text{A}, p_\text{B}, p_\text{C}, p_\text{D}, \text{Sens}_0, \text{Sens}_\text{A}, \text{Sym}_\text{A}).
$$

Therefore by the definition of the maximum likelihood estimator,

$$
\begin{aligned}
(\widehat{p}_0, \widehat{p}_\text{A}, \widehat{p}_\text{B}, \widehat{p}_\text{C}, \widehat{p}_\text{D}, \widehat{\text{Sens}}_0, \widehat{\text{Sens}}_\text{A}, \widehat{\text{Sym}}_\text{A}) :=& \operatorname*{arg\,min}_{p_0, p_\text{A}, p_\text{B}, p_\text{C}, p_\text{D}, \text{Sens}_0, \text{Sens}_\text{A}, \text{Sym}_\text{A}} -\log \mathcal{L} \\
=& -\log[\mathcal{L}_{m_0}(p_0, p_\text{A}, \dots, p_\text{D}) + \dots + \mathcal{L}_{m_\text{D}}(p_\text{D}) \\
&+ \mathcal{L}_{D_0, D_\text{A}}(p_0, p_\text{A}, \text{Sens}_0, \text{Sens}_\text{A}) + \mathcal{L}_{D_\alpha}(p_\text{A}, p_\text{B}, \text{Sym}_\text{A})],
\end{aligned}
$$

which was numerically solved using the Nelder-Mead method. [6]

[4] This was chosen by first solving the MLE with $\text{Sym}_\text{A} = 0$ and then $\text{Sym}_\text{B} = 0$ and considering the midpoint of these two solutions. The midpoint sits at approximately $8 \times \text{Sym}_\text{A} = \text{Sym}_\text{B}$.

2.1.4 Bootstrap Resamples

The bootstrap samples $m_0^{*b}, m_{\mathrm{A}}^{*b}, m_{\mathrm{B}}^{*b}, m_{\mathrm{C}}^{*b}, m_{\mathrm{D}}^{*b}, D_0^{*b}, D_{\mathrm{A}}^{*b}, D_\alpha^{*b}$ for $b \in \{1, 2, \ldots, 10000\}$ were generated as follows:

1. m_X^{*b} was the median value of n_X samples from a $\mathrm{HypoGeom}(\widehat{p}_{\mathrm{D}}, \widehat{p}_{\mathrm{C}}, \ldots, \widehat{p}_X)$.

2. $$D_0^{*b} \sim \mathrm{Binom}\left(k, \frac{\widehat{\mathrm{Sens}_0}(1-(1-\widehat{p}_0)^6)}{6 \times \widehat{p}_0(1-(1-\widehat{\mathrm{Sens}_0})(1-\widehat{p}_0)^6)}\right).$$

3. $$D_{\mathrm{A}}^{*b} \sim \mathrm{Binom}\left(k - D_0^{*b}, \frac{\mathbf{t}(\widehat{p}_0, \widehat{p}_{\mathrm{A}}, \widehat{\mathrm{Sens}_0}, \widehat{\mathrm{Sens}_{\mathrm{A}}})_2}{1 - \mathbf{t}(\widehat{p}_0, \widehat{p}_{\mathrm{A}}, \widehat{\mathrm{Sens}_0}, \widehat{\mathrm{Sens}_{\mathrm{A}}})_1}\right).$$

4. $$D_\alpha^{*b} \sim \mathrm{Binom}\left(\bar{s}, \left(1 - \frac{\widehat{\mathrm{Sym}_{\mathrm{A}}}}{1-(1-\widehat{p}_{\mathrm{A}})\widehat{\mathrm{Sym}_{\mathrm{A}}}}\right) \times \frac{8 \times \widehat{\mathrm{Sym}_{\mathrm{A}}}}{1 - 8(1-\widehat{p}_{\mathrm{B}}) \times \widehat{\mathrm{Sym}_{\mathrm{A}}}} + \frac{\widehat{\mathrm{Sym}_{\mathrm{A}}}}{1-(1-\widehat{p}_{\mathrm{A}})\widehat{\mathrm{Sym}_{\mathrm{A}}}}\right).$$

2.1.5 Bootstrap Maximum Likelihood Estimates

Then as above, letting

$$\mathcal{L}^{*b} := \mathcal{L}_{m_0^{*b}, m_{\mathrm{A}}^{*b}, m_{\mathrm{B}}^{*b}, m_{\mathrm{C}}^{*b}, m_{\mathrm{D}}^{*b}, D_0^{*b}, D_{\mathrm{A}}^{*b}, D_\alpha^{*b}}(p_0, p_{\mathrm{A}}, p_{\mathrm{B}}, p_{\mathrm{C}}, p_{\mathrm{D}}, \mathrm{Sens}_0, \mathrm{Sens}_{\mathrm{A}}, \mathrm{Sym}_{\mathrm{A}}),$$

each

$$(\widehat{p}_0^{*b}, \widehat{p}_{\mathrm{A}}^{*b}, \widehat{p}_{\mathrm{B}}^{*b}, \widehat{p}_{\mathrm{C}}^{*b}, \widehat{p}_{\mathrm{D}}^{*b}, \widehat{\mathrm{Sens}_0}^{*b}, \widehat{\mathrm{Sens}_{\mathrm{A}}}^{*b}, \widehat{\mathrm{Sym}_{\mathrm{A}}}^{*b}) := \underset{p_0, p_{\mathrm{A}}, p_{\mathrm{B}}, p_{\mathrm{C}}, p_{\mathrm{D}}, \mathrm{Sens}_0, \mathrm{Sens}_{\mathrm{A}}, \mathrm{Sym}_{\mathrm{A}}}{\arg\min} - \log \mathcal{L}^{*b}$$

was numerically solved using the Nelder-Mead method.

2.2 Bootstrap Results

The results from the bootstrapped data, and bootstrap maximum likelihood estimates can be seen in Supplementary Figures S1 and S2.

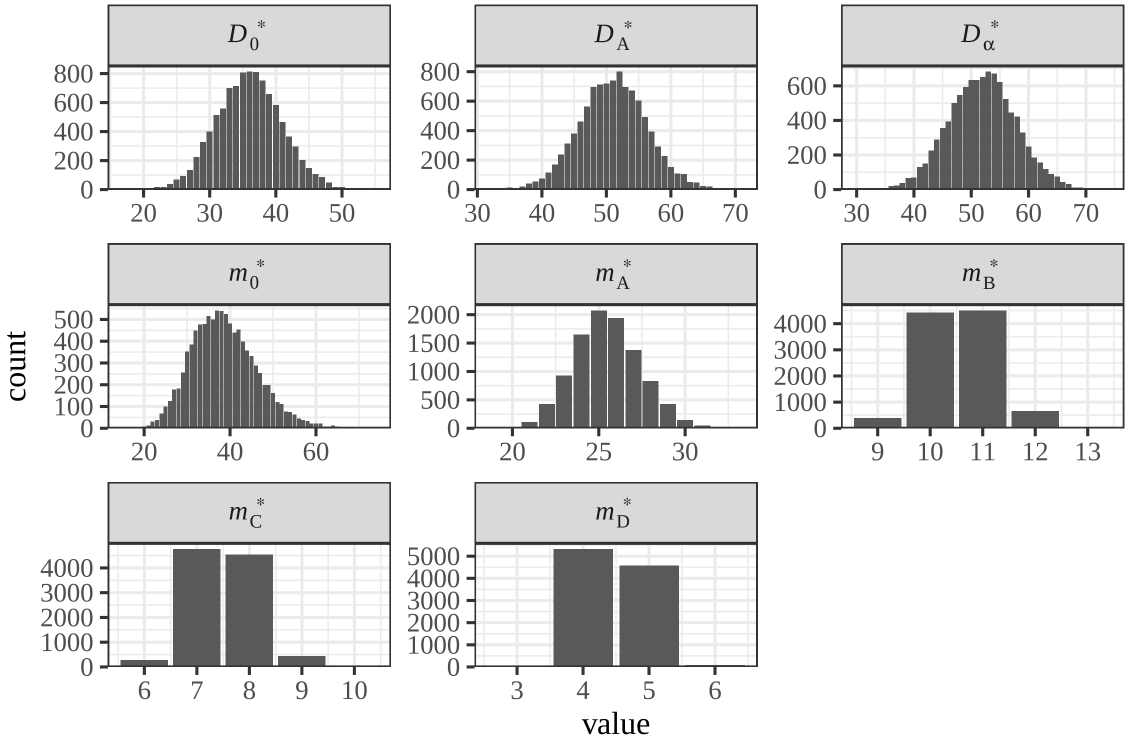

Supplementary Figure S1: The variation in the bootstrap samples of the data.

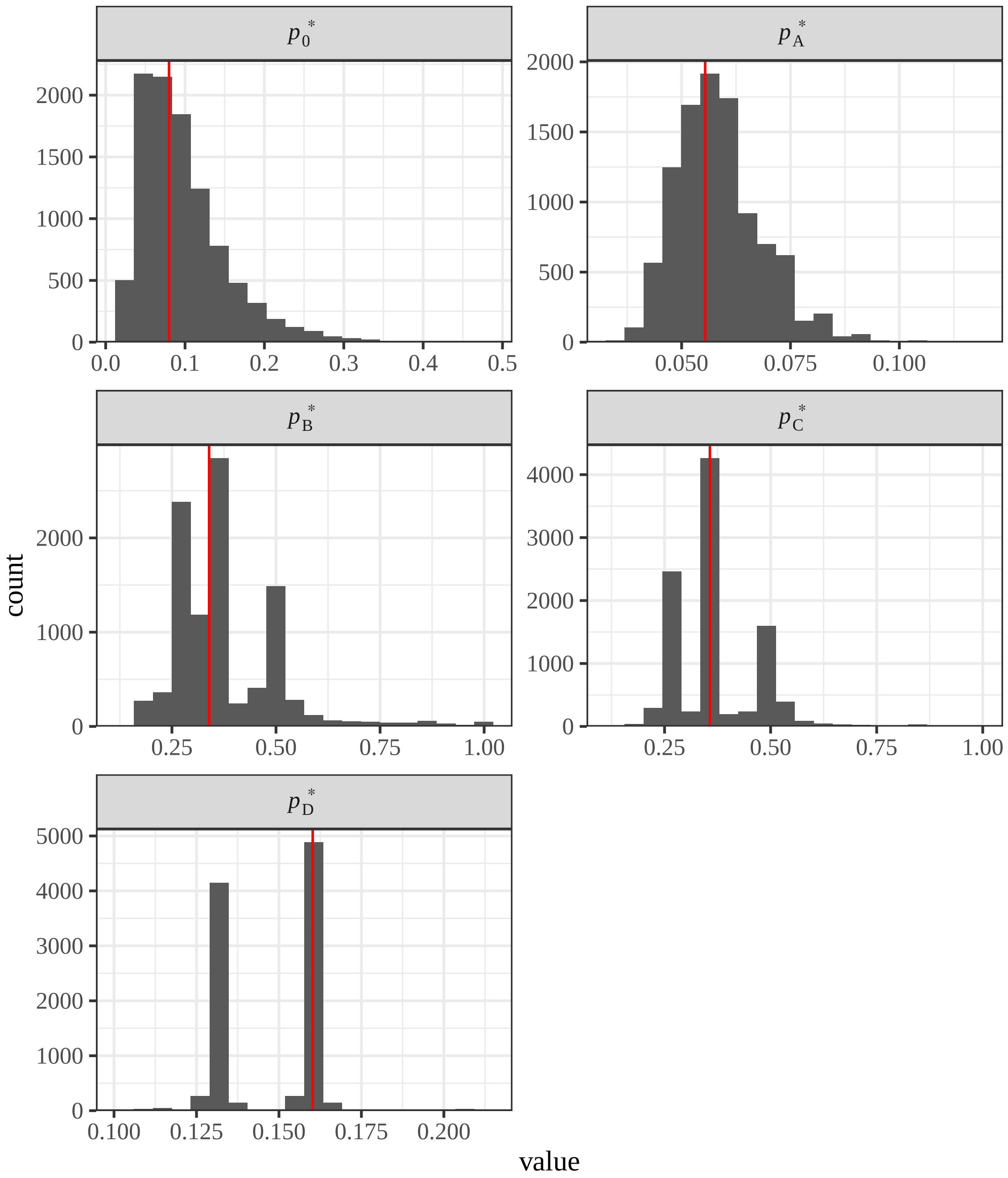

Supplementary Figure S2: The variation in the bootstrap maximum likelihood estimates of transition probabilities. The multimodal nature of the estimates is due to the discrete values that the m_X^*'s could take. This is particularly strong in p_D, since m_D^* was almost entirely either 4 or 5. Red lines are the MLEs from the true data.

Let B be the true baseline output. This can be estimated by $\widehat{B}$, using the maximum likelihood estimates from the true data calculated above, and using the point estimates from the data estimated. B^{*b} is the model estimate using the bth set of maximum likelihood parameters calculated as above. The estimated standard error of $\widehat{B}$ was 5.7 (see Supplementary Figure S3).

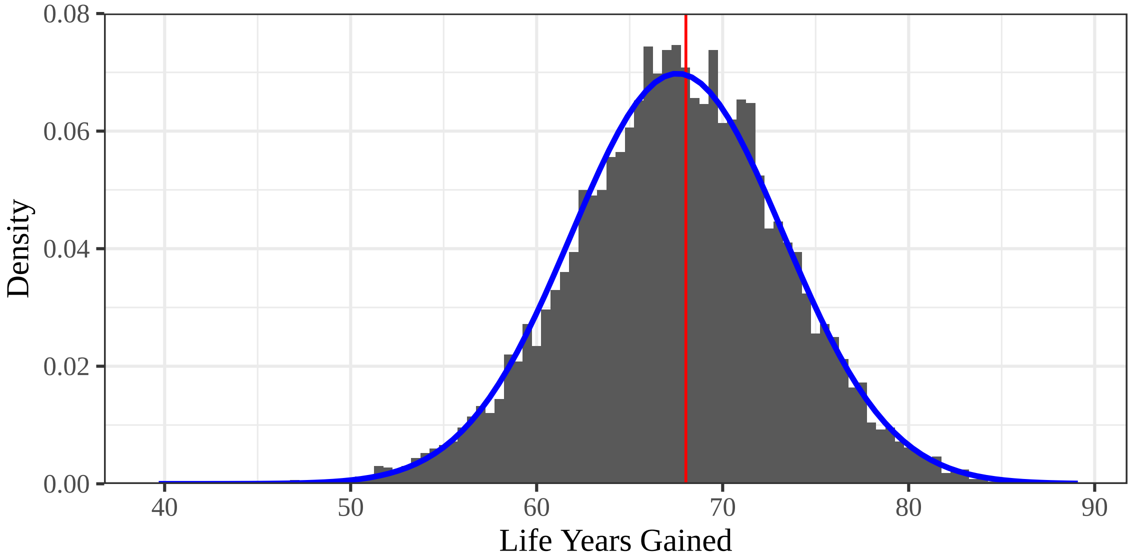

Supplementary Figure S3: The variation in B^*. The blue line is a fitted normal distribution with $\mathcal{N}(\mu = 68.0, \sigma^2 = (5.7)^2)$. The red line is $\widehat{B}$.

3 Adherence Data

3.1 Adherence to Surveillance Data Methods

We derived the model adherence parameters from empirical data taken from a clinical hepatocellular carcinoma database in a large tertiary referral hepatocellular carcinoma centre in the Australia between 2003 and 2022. Patient ultrasound dates were extracted from the liver clinic database for all patients with a record of hepatitis C related cirrhosis. Hepatocellular carcinoma diagnosis date, death (yes/no and date) and loss to follow up (yes/no and date) were extracted from the hepatitis C or hepatocellular carcinoma multidisciplinary clinic database for all patients with a hepatitis C or hepatocellular carcinoma diagnosis. Death and loss to follow-up data were not available for all patients. Patient data was matched using patient identifiers provided in each data set. Those who were recorded as being without cirrhosis and/or without an sustained virologic response prior to hepatocellular carcinoma diagnosis were excluded from the analysis. If no sustained virologic response date was available, sustained virologic response was defined to be 12 weeks after direct-acting antivirals completion. If this wasn't available, then for patients who were identified as having achieved sustained virologic response, the sustained virologic response date was defined as the latter of six months before the first recorded ultrasound, or the 1st of July 2016 (including those with recorded ultrasounds prior to widespread administration of direct acting antivirals in 2016). Patients were censored at the date of death, hepatocellular carcinoma diagnosis, or the 1st of March 2020 to control for the effect of Covid-19 on ultrasound surveillance adherence rates. The expected number of ultrasounds was calculated as the number of months since sustained virologic response to time of death/censor divided by six, always rounding down (so that someone who had achieved sustained virologic response 18-23 months prior to censor data would have 3 expected ultrasounds). The adherence results were also compared to results that included data until the database close date on the 18th of March 2022.

The ratio of actual to expected ultrasounds (based on six-monthly surveillance) for each patient was defined as their attendance rate. In order to obtain a reliable indicator of adherence, the cohort was restricted to those with an estimated sustained virologic response 18 months prior to the 1st March 2020 (or 18 months before hepatocellular carcinoma diagnosis/death) to allow sufficient follow up for at least three expected ultrasounds before the censor date. Those who had a less than 10% adherence rate (ie equivalent to undergoing one ultrasound every five years) were considered "non-adherent" and those with a greater than 80% adherence rate (equivalent to missing one ultrasound every 2.5 years) were considered "completely adherent". The population-level average attendance rate amongst those considered "partially adherent" was defined as total ultrasounds attended among partially adherent divided by total expected ultrasounds among partially adherent. Only non-adherent and completely adherent cohort parameters are specified in the model, since the probability of being in the partially adherent cohort can be calculated directly by $\Pr(\text{Partially adherent}) = 1 - \Pr(\text{Non-adherent}) - \Pr(\text{Completely adherent})$. These definitions were informed by the trends in of the data (see Supplementary Figure S4).

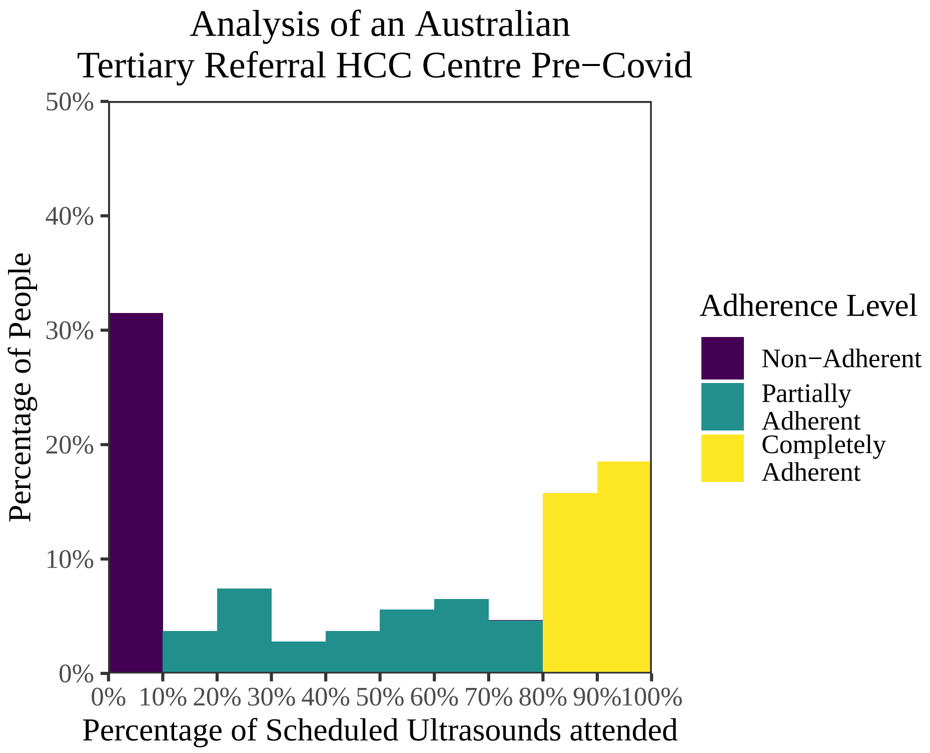

Supplementary Figure S4: Analysis results for the adherence of those with cirrhosis and having being cured of hepatitis C in a tertiary hospital liver clinic pre-covid.

3.2 Adherence to Surveillance Data Results

Of 218 patients attending an Australian tertiary hospital liver clinic pre-covid, 108 (50%) were eligible for inclusion. The median period of observation was 3.2 years (IQR: 0.3 years), and the median number of ultrasounds was 3 (IQR: 5). Adherence was skewed to the extremes, particularly towards non-adherence. 31.5% of the population were non-adherent, 34.3% were partially adherent, and 34.3% were completely adherent to surveillance recommendations. Of those who were partially adherent, they attend 44.9% of appointments on average. Overall adherence was 47%.

4 Effect of Change in Adherence Probability

In order to identify whether increasing the probability ultrasound attendance has diminishing returns, we simulated multiple populations with varying probabilities of attending scheduled ultrasound surveillance. For this analysis, within a population, each person had equal probability of attending scheduled ultrasound surveillance.

We considered all possible values of adherence probabilities (0%-100%). Although there is some drop-off to the intervention gains per percentage point increase in adherence as adherence rate increases, small increases in adherence rate still result in significant gains close to complete adherence as seen in Supplementary Figure S5.

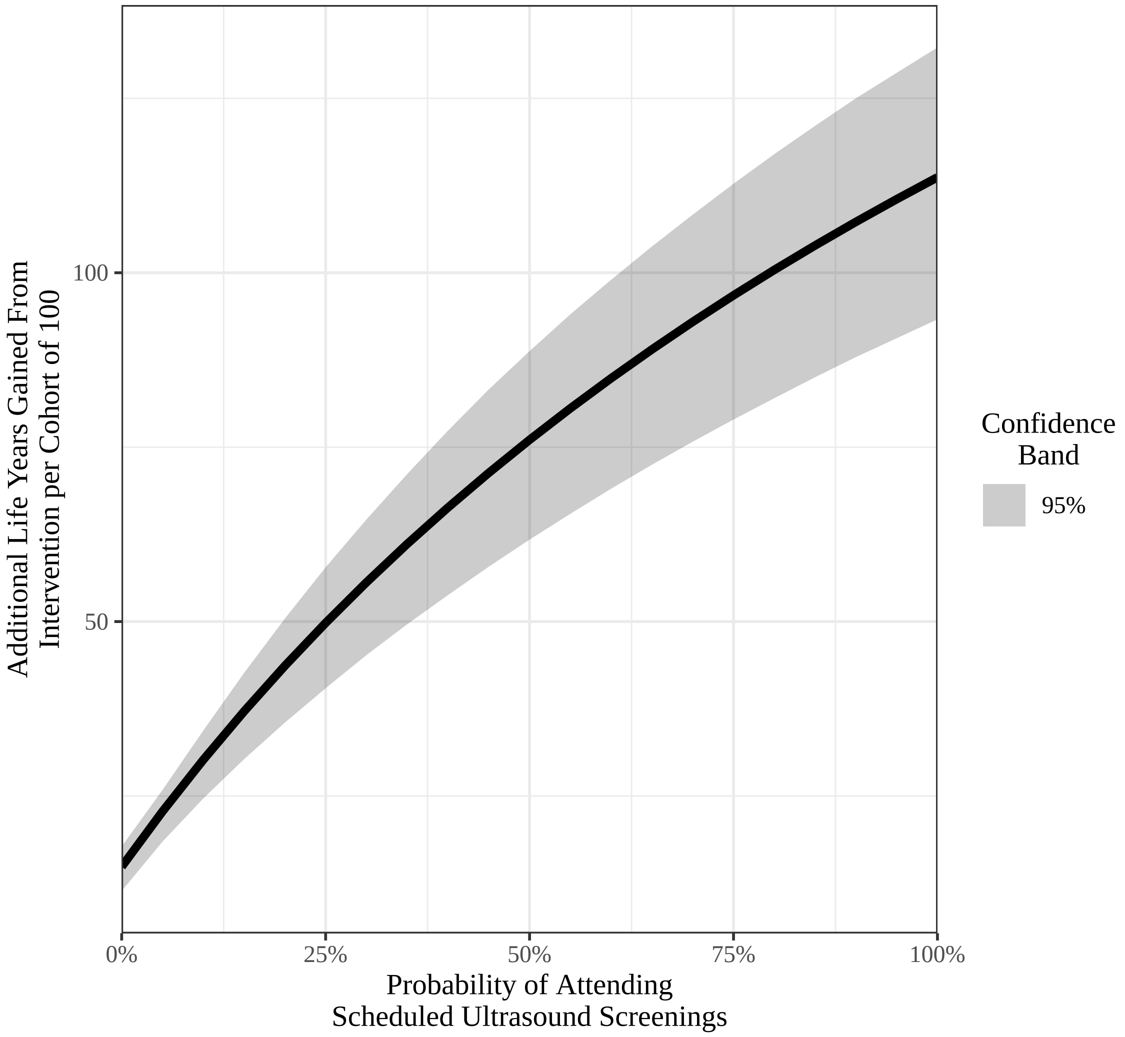

Supplementary Figure S5: The effect of varying the probability of adherence to surveillance on life years gained in a simulation of 100 people over 10 years. Each simulation population had the a uniform probability of adherence.

5 Heterogeneity in Risk of HCC

In order to determine whether the rate at which someone develops HCC impacts the effectiveness of interventions, we simulated the scenarios under a low and high risk of developing HCC (half and double the assumed risk used in the baseline model). The absolute sizes of the effect o f the interventions changed proportionate to the rate of developing HCC, but the comparative effects were unchanged (with adherence to ultrasound screening recommendations being the most impactful) as seen in Suplementary Figure S6 and Suplementary Figure S7.

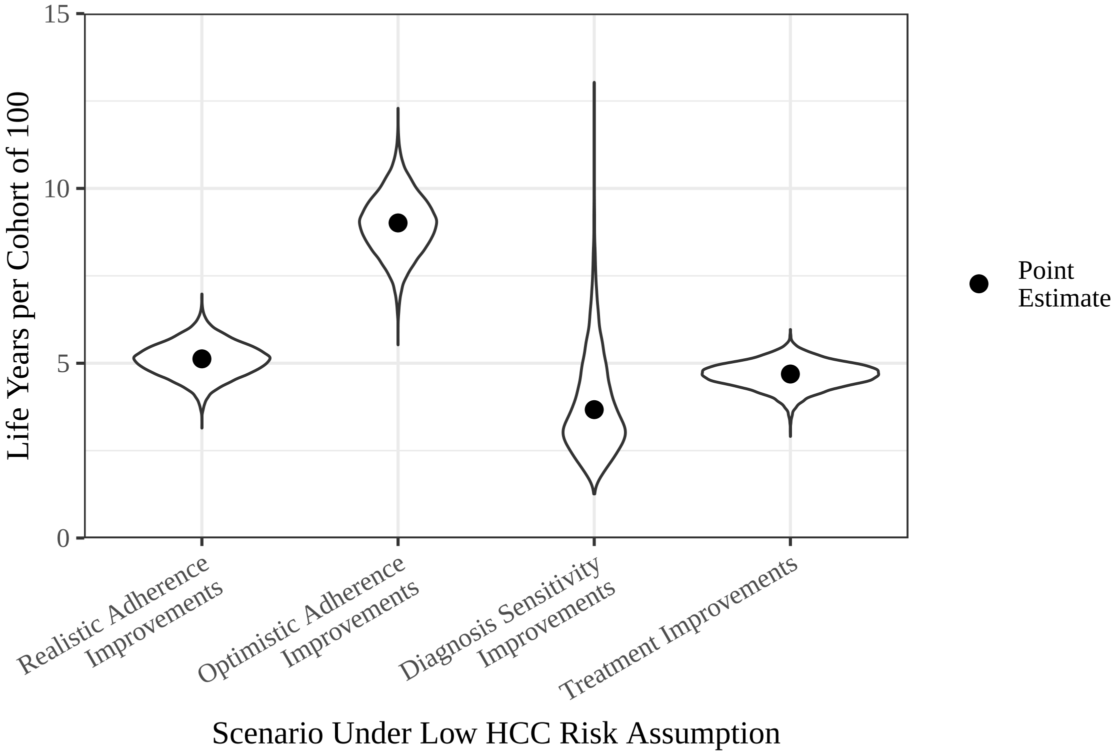

Supplementary Figure S6: Over a 10 year simulated intervention with 100 people, this figure shows how four scenarios effect the mean additional years of life that are attributable to each diagnosed case given a low annual risk of developing HCC (1% per year).

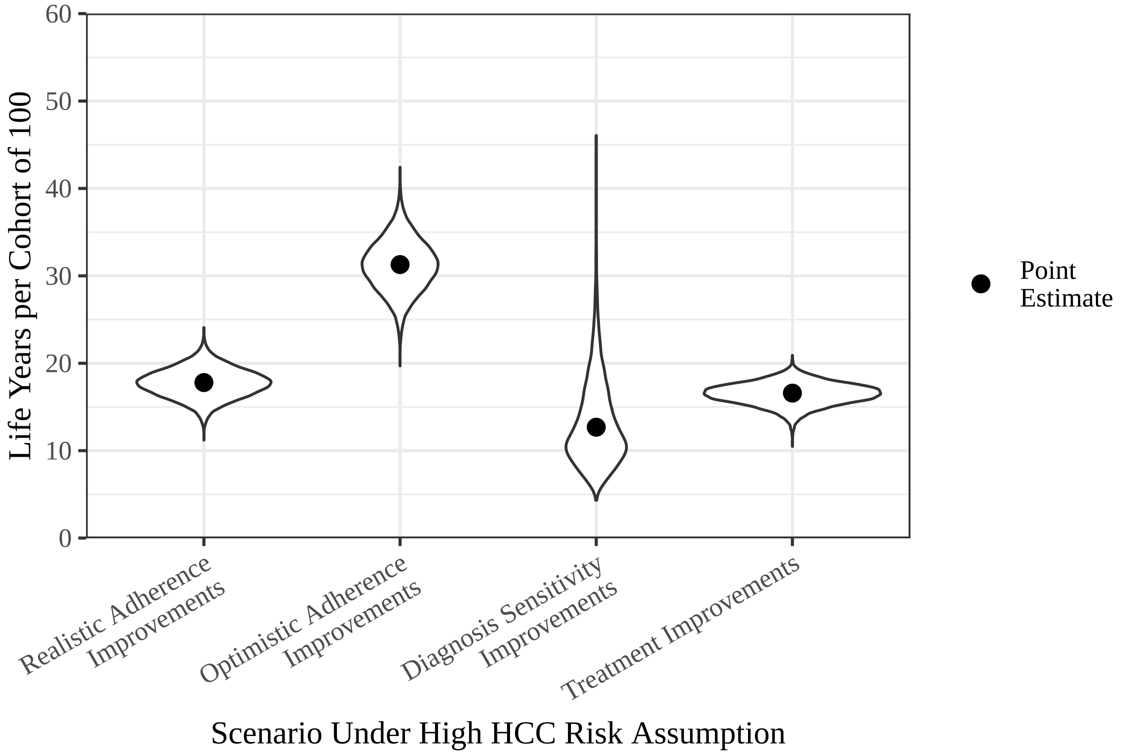

Supplementary Figure S7: Over a 10 year simulated intervention with 100 people, this figure shows how four scenarios effect the mean additional years of life that are attributable to each diagnosed case given a high annual risk of developing HCC (4% per year).

6 Transition Matrix

$$\begin{bmatrix}
1 & 0 & 0 & 0 & 0 & 0 & 0 & 0 & 0 & 0 & 0 & 0 & 0 & 0 & 0 \\
0 & 1 & 0 & 0 & 0 & 0 & 0 & 0 & 0 & 0 & 0 & 0 & 0 & 0 & 0 \\
0 & 0 & 0 & 1-p_0 & 0 & 0 & 0 & 0 & 0 & p_0 & 0 & 0 & 0 & 0 & 0 \\
0 & 0 & 0 & 0 & 1-p_0 & 0 & 0 & 0 & 0 & 0 & p_0 & 0 & 0 & 0 & 0 \\
0 & 0 & 0 & 0 & 0 & 1-p_0 & 0 & 0 & 0 & 0 & 0 & p_0 & 0 & 0 & 0 \\
0 & 0 & 0 & 0 & 0 & 0 & 1-p_0 & 0 & 0 & 0 & 0 & 0 & p_0 & 0 & 0 \\
0 & 0 & 0 & 0 & 0 & 0 & 0 & 1-p_0 & 0 & 0 & 0 & 0 & 0 & p_0 & 0 \\
(1-p_0)\mathrm{Sens}_0 & p_0\times\mathrm{Sens}_\mathrm{A} & (1-p_0)(1-\mathrm{Sens}_0) & 0 & 0 & 0 & 0 & 0 & p_0(1-\mathrm{Sens}_\mathrm{A}) & 0 & 0 & 0 & 0 & 0 & 0 \\
0 & 0 & 0 & 0 & 0 & 0 & 0 & 0 & 0 & (1-p_\mathrm{A}) & 0 & 0 & 0 & 0 & p_\mathrm{A} \\
0 & 0 & 0 & 0 & 0 & 0 & 0 & 0 & 0 & 0 & (1-p_\mathrm{A}) & 0 & 0 & 0 & p_\mathrm{A} \\
0 & 0 & 0 & 0 & 0 & 0 & 0 & 0 & 0 & 0 & 0 & (1-p_\mathrm{A}) & 0 & 0 & p_\mathrm{A} \\
0 & 0 & 0 & 0 & 0 & 0 & 0 & 0 & 0 & 0 & 0 & 0 & (1-p_\mathrm{A}) & 0 & p_\mathrm{A} \\
0 & 0 & 0 & 0 & 0 & 0 & 0 & 0 & 0 & 0 & 0 & 0 & 0 & (1-p_\mathrm{A}) & p_\mathrm{A} \\
0 & (1-p_\mathrm{A})\mathrm{Sens}_\mathrm{A} & 0 & 0 & 0 & 0 & 0 & 0 & (1-p_\mathrm{A})(1-\mathrm{Sens}_\mathrm{A}) & 0 & 0 & 0 & 0 & 0 & p_\mathrm{A}
\end{bmatrix} \tag{S1}$$

References

[1] Edoardo G. Giannini, Fabio Farinati, Francesca Ciccarese, Anna Pecorelli, Gian Lodovico Rapaccini, Mariella Di Marco, Luisa Benvegnù, Eugenio Caturelli, Marco Zoli, Franco Borzio, Maria Chiaramonte, Franco Trevisani, and for the Italian Liver Cancer group. Prognosis of untreated hepatocellular carcinoma. *Hepatology*, 61(1):184–190, 2015. doi: 10.1002/hep.27443.

[2] Korosh Khalili, Ravi Menezes, Tae Kyoung Kim, Leyla Kochak Yazdi, Hyun-Jung Jang, Suraj Sharma, Jordan Feld, and Morris Sherman. The Effectiveness of Ultrasound Surveillance for Hepatocellular Carcinoma in a Canadian Centre and Determinants of Its Success. *Canadian Journal of Gastroenterology and Hepatology*, 29:563893, 2015. doi: 10.1155/2015/563893.

[3] T. P. Hong, P. J. Gow, M. Fink, A. Dev, S. K. Roberts, A. Nicoll, J. S. Lubel, I. Kronborg, N. Arachchi, M. Ryan, W. W. Kemp, V. Knight, V. Sundararajan, P. Desmond, A. J. Thompson, and S. J. Bell. Surveillance improves survival of patients with hepatocellular carcinoma: a prospective population-based study. *Med J Aust*, 209(8):348–354, 2018. doi: 10.5694/mja18.00373.

[4] Stefanka Chukova, Leda Minkova, and Silvana Paralloi. Hypogeometric distribution and related discrete time point process. *Istatistik Journal of The Turkish Statistical Association*, 14(1):1–10, 2022. Publisher: Başbakanlık.

[5] Andri Signorell. *DescTools: Tools for Descriptive Statistics*, 2023. URL `https://CRAN.R-project.org/package=DescTools`. R package version 0.99.52.

[6] J. A. Nelder and R. Mead. A simplex method for function minimization. *The Computer Journal*, 7(4):308–313, 1965. ISSN 0010-4620. doi: 10.1093/comjnl/7.4.308. URL `https://doi.org/10.1093/comjnl/7.4.308`.

[7] R Core Team. *R: A Language and Environment for Statistical Computing*. R Foundation for Statistical Computing, Vienna, Austria, 2023. URL `https://www.R-project.org/`.

[8] Seongho Kim. *ppcor: Partial and Semi-Partial (Part) Correlation*, 2015. URL `https://CRAN.R-project.org/package=ppcor`. R package version 1.1.

[9] Damian Pavlyshyn. *hypogeom: Distribution Functions For the Hypogeometric Distribution*, 2023. R package version 0.0.0.9000.

[10] Horst Lewitschnig and David Lenzi. *GenBinomApps: Clopper-Pearson Confidence Interval and Generalized Binomial Distribution*, 2022. URL `https://CRAN.R-project.org/package=GenBinomApps`. R package version 1.2.
